# Environmentally Friendly Hybrid Organic–Inorganic Halogen-Free Coatings for Wood Fire-Retardant Applications

**DOI:** 10.3390/polym14224959

**Published:** 2022-11-16

**Authors:** Georgia C. Lainioti, Vasilis Koukoumtzis, Konstantinos S. Andrikopoulos, Lazaros Tsantaridis, Birgit Östman, George A. Voyiatzis, Joannis K. Kallitsis

**Affiliations:** 1 Foundation for Research and Technology-Hellas, Institute of Chemical Engineering Sciences (FORTH/ICE-HT), Stadiou Str, GR–26504 Rio-Patras, Greece; 2 Department of Chemistry, University of Patras, GR-26504 Patras, Greece; 3 Department of Physics, University of Patras, GR-26504 Patras, Greece; 4 RISE, Drottning Kristinas väg 61, P.O. Box 5604, SE-11486 Stockholm, Sweden; 5 Department of Building and Energy Technology, Linnaeus University, P.O. Box 451, SE-35106 Växjö, Sweden

**Keywords:** fire retardants, polymeric coatings, wood applications, reactive oligomers, Euroclass

## Abstract

Wood and wood-based products are extensively used in the building sector due to their interesting combination of properties. Fire safety and fire spread, however, are of utmost concern for the protection of buildings. Therefore, in timber structures, wood must be treated with fire-retardant materials in order to improve its reaction to fire. This article highlights the flame retardancy of novel hybrid organic–inorganic halogen-free coatings applied on plywood substrates. For this purpose, either a huntite-rich mineral (H5) or its modified nano-Mg (OH)_2_ type form (H5-m), acting as an inorganic (nano) filler, was functionalized with reactive oligomers (ROs) and incorporated into a waterborne polymeric matrix. A water-soluble polymer (P (SSNa-co-GMAx)), combining its hydrophilic nature with functional epoxide groups, was used as the reactive oligomer in order to enhance the compatibility between the filler and the matrix. Among various coating compositions, the system composed of 13% polymeric matrix, 73% H5 and 14% ROs, which provided the best coating quality and flame retardancy, was selected for the coating of plywood on a larger scale in one or two layers. The results indicated that the novel plywood coating systems with the addition of ecological coating formulations (WF-13, WF-14 and WF-15), prepared at two layers, reached Euroclass B according to EN13501-1, which is the best possible for fire systems applied to wood.

## 1. Introduction

Wood-based products are widely used as building materials since they exhibit a variety of performance requirements [1,2]. They are renewable and sustainable materials that have gained great attention in many value-added applications due to their excellent properties. Good strength and durability, electrical and heat resistance, sound absorption, aesthetic properties, low environmental pollution and health aspects are some of the advantages of wood that justify the use of wood materials for construction (both new buildings and restoration processes) and furniture [3,4].

However, fire performance is one of the main obstacles to the increased use of wood in most countries. New European classification systems for fire performance have been developed, but national safety levels still remain, causing continued limitations in the use of wood products. For the reduction in flammability, wood products are treated with flame retardants (FRs) that reduce or eliminate the risk of fire [5,6]. The commercial market for these additives is presently dominated by compounds containing halogens, such as brominated flame retardants (BFRs) [7], which offer a high standard of flame retardancy and exhibit sufficient physical properties. Moreover, the use of halogenated compounds leads to an increase in the amount of smoke and toxic decomposition products formed during the combustion process, which is extremely burdensome for human survival and the surrounding environment, threatening both air and aquatic ecosystems [8,9,10]. Hence, there is an urgent need for the design of new environmentally friendly FR materials to substitute/eliminate the use of halogenated FRs [11,12]. It is worth noting that there are countries in Europe that have already issued regulations for the restriction of brominated flame retardants by enhancing the use of harmless flame retardants, with quite a high efficiency and low toxicity [13,14].

Wood combustion proceeds initially by water evaporation followed by a temperature increase to about 150 °C at which the degassing begins with the formation of flammable degradation products combined with flame formation [15]. To control that process, the FR additives should either enhance the pyrolysis rate, resulting in char formation, or form an isolating layer that reduces the release of pyrolysis gases and access to oxygen on the surface, thus delaying the temperature rise. Among relevant additives, inorganic FRs, such as magnesium hydroxide (MH), are very promising substitutions for the replacement of BFRs. These materials provide flame-retardant formulations that lead to combustion products with low opacity, low toxicity and nominal corrosivity that are easy to handle [16]. Magnesium hydroxide constitutes an effective flame retardant and harmless filler that has been widely used over the past few decades in polymeric materials due to its high decomposition temperature, which allows its use in wood materials processed at temperatures close to 350 °C [17,18]. In addition to MH, a number of minerals showing flame-retardant properties that could be of potential benefit in polymers have been identified. Hydromagnesite is naturally occurring in mixtures with huntite. The onset of decomposition for hydromagnesite is at ~250 °C and ends slightly above 450 °C, while the corresponding range for huntite is at higher temperatures. These temperatures are higher than that of MH as well as of other metal hydroxides, making the above-mentioned minerals more suitable for polymer composites prepared by melt blending, where high processing temperatures are usually required.

Since the inorganic flame retardants mentioned above are insoluble in the polymer matrices to which they are added, functionalization by coupling agents may enhance the compatibility between the filler and the matrix, avoiding undesirable effects such as water pick up and swell, poor dispersion and distribution and a reduction in the physical properties of the composites. A very broad new family of compounds, the reactive oligomers (ROs), presents multiple advantages as coupling agents in comparison to traditional ones [19,20]. They can be easily adapted to fit a specific application, and their ability to bind chemically to Mg-based inorganic particles is remarkable. This, eventually, can lead to direct functionalization at the processing line without additional steps of external chemical modification.

In the present work, novel flame-retardant systems were developed based on inorganic materials modified with ROs as coupling agents with the aim of enhancing the compatibility between the fillers and the matrices. More specifically, epoxide reactive oligomer poly (sodium 4-styrenesulfonate-co-glycidyl methacrylate) (P (SSNa-co-GMAx)) [21,22] was dispersed in water and then Mg-based particles and the water-based polymer matrix (Vilacryl DS55K) were subsequently added in order to create coatings in various mixture compositions. The novelty of the work consists of the efficient combination of water-soluble functional copolymers with a high amount of inorganic fillers providing high-quality flame-retardant wood coatings. Based on this idea, different coating formulations were developed for wood materials for construction and/or furniture to be applied both in new products and in restoration processes. Relevant coatings were subsequently tested in terms of their reaction-to-fire testing, and additionally, their fire classes were determined according to the European system with Euroclass classification EN 13501-1. The flame-retardant coatings designed in this work will lead to an innovative group of coatings for wood treatment to be used in newly manufactured as well as restored products.

## 2. Materials and Methods

### 2.1. Materials

The monomers glycidyl methacrylate (GMA), sodium 4-styrene sulfonate (SSNa) and the initiator azobisisobutyronitrile (AIBN), as well as deuterium oxide (D_2_O), were purchased from Aldrich (Aldrich, Steinheim, Germany) and used as received. The solvents dimethyl sulfoxide (DMSO) and acetone were purchased from Fischer Scientific (Fisher Scientific, Pittsburgh, PA, USA) and used as received. The water-based polymer matrix Vilacryl DS55K is a styrene acrylic copolymer MFFT~2 and was provided by LOUFAKIS CHEMICALS S.A. (Thessaloniki, Greece). Ultra-pure water was obtained by means of an SG apparatus water purification unit.

### 2.2. Copolymer Synthesis

**Scheme 1:**
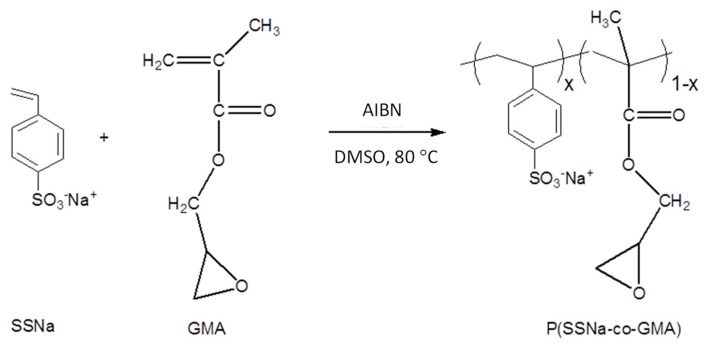
Synthesis of P (SSNa-co-GMA20).

The copolymer poly (sodium styrene sulfonate-co-glycidyl methacrylate) was synthesized through free radical polymerization using AIBN as the initiator according to an experimental procedure previously described [21,23]. The solvent used for the reaction was DMSO. The desired quantities of the two monomers (total monomer concentration 1 M) were dissolved in DMSO, the solution was degassed, and the initiator AIBN (0.8 mol % over the total monomer concentration) was added. The reaction was left to proceed overnight under vigorous stirring in an Ar atmosphere in an oil bath set at 80 °C. The experimental procedure is shown in Scheme 1. After cooling down to room temperature, the copolymers were recovered by precipitation in acetone, filtered and dried in a vacuum oven at 60 °C for 24 h. The mol fraction of GMA was calculated either through ^1^H NMR spectroscopy, using D_2_O as a solvent, or thermogravimetric analysis (TGA), as reported in previous work [22]. The synthesized copolymers represent the reactive oligomers incorporated into the flame-retardant coatings and are denoted as P (SSNa-co-GMA20), where 20 is the %mol fractions of GMA units as determined by the ^1^H NMR characterization in D_2_O.

### 2.3. Huntite Modification

The huntite (H5) mineral was kindly provided by Sibelco Hellas SA and consisted of a huntite/hydromagnesite mixture, 88%/12%; the ratio was calculated by neglecting minor concentrations of other minerals, mostly dolomite. H5 was subjected to thermal treatment at 500 or 550 °C for 1 h in ambient air [18]. The powders after calcination (4 g), consisting primarily of oxides (mostly MgO as well as CaO), were hydrated by dispersing in distilled water (40 mL) and stirring at 90 °C for 4 h. In this way, nano-Mg (OH)_2_ was formed according to the following reaction:

2MgO + 2H_2_O → 2Mg(OH)_2_ (1)

Then, its aqueous dispersion was centrifuged at 8500 rpm for 8 min. The final product, named H5-m, was collected after drying the hydrated product in the oven at 90 °C for 20 h.

### 2.4. Blend Preparation

Blends of polymer matrix VILACRYL DS55K, huntite H5 or H5-m and the RO P (SSNA-co-GMA20) were prepared in diverse ratios. Precisely, the copolymer P (SSNA-co-GMA20) was dissolved in ultra-pure water at a concentration of 10% *w*/*v*. After complete dissolution of the copolymer, either H5 or H5-m was added, and the system was left until complete homogenization. The last step was the addition of the polymer matrix VILACRYL DS55K. The system was finally placed in an ultrasonic bath for 5 min for the dispersion of the final mixture.

### 2.5. Characterization of Surfaces/Prepared Mixtures on Plywood or Wood Substrate

The morphology of the materials was studied by a scanning electron microscope (SEM) Zeiss SUPRA 35 VP-FEG (Carl Zeiss, Germany) equipped with energy-dispersive X-ray spectroscopy (EDS) (Bruker GmbH, Quanta200, Berlin, Germany) and backscattered electron (BSE) detectors (K.E. Developments, Ltd., Cambridge, U.K.), operating at 5–20 keV.

#### 2.5.1. Limited Oxygen Index (LOI) Measurements

The value of LOI is defined as the minimum concentration of oxygen in the oxygen/nitrogen mixture [O_2_/N_2_] that either maintains the flame burn of a material for 3 min or consumes 5 cm of sample length, with the sample placed in a vertical position (flame is applied at the top of the specimen).

LOI is expressed as: LOI = 100 ([O_2_]/([O_2_] + [N_2_])).

The specimens used for the test of reaction-to-fire performance were 70 mm (length) × 6.5 mm (width) × 3 mm (thickness). For their preparation, a press was used in which various pressures and temperatures were applied [24,25].

#### 2.5.2. Wood Products and Fire Test Results

Birch plywood, 9 mm, complying with EN 13238 [26] and with minimum knots in the surface layer (since knots may influence the test results at small-scale testing) was chosen as the standard substrate for reaction-to-fire testing. Suitable quality is grade S according to EN 635 at the surface to be coated and tested; the back side may have lower quality, e.g., grade BB with more visible knots.

**Figure 1:**
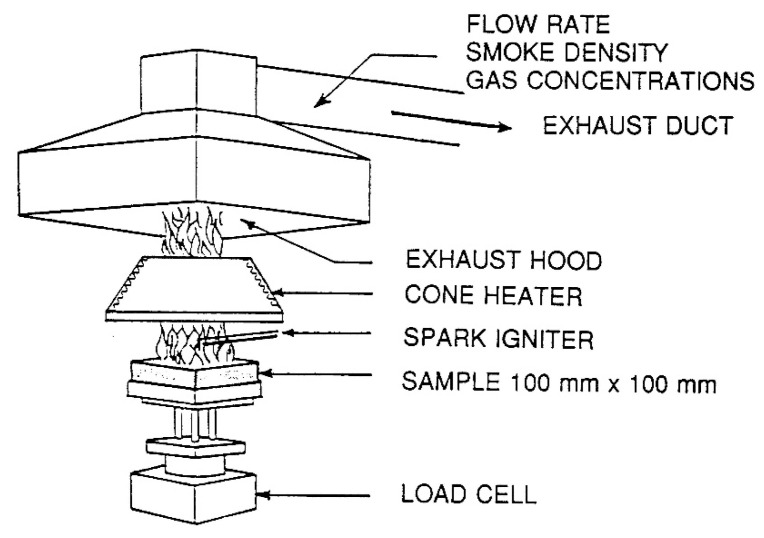
Fire testing using the cone calorimeter ISO 5660-1 (a schematic picture).

Reaction-to-fire testing was performed according to the cone calorimeter ISO 5660-1 [27] at the heat flux 50 kW/m^2^ and complemented by predictions of possible reaction-to-fire classes according to the European system [28]. Fifteen products, including two controls without any coating, were tested according to this procedure. The main parameters determined in the cone calorimeter are heat release rate (HRR) and time to ignition (TTI). The cone calorimeter used is a commercially available apparatus from FTT (Fire Testing Technology Ltd., East Grinstead, UK). Based on the cone calorimeter results, the possible European reaction-to-fire class could be predicted according to a model already published [29,30]. The fire testing using the cone calorimeter ISO 5660-1 is shown in Figure 1.

Specimens were prepared in order to facilitate a systematic evaluation of test results, i.e., only one parameter was changed at a time for each type of coating. Important parameters were the different compositions and amounts of components in the coating and the different amounts of each coating (g/m^2^).

Three levels of coating were used when studying each parameter. The possible influence and reaction-to-fire class could then be predicted by interpolation. The specimen size for each type and/or level of coating was about A4 size (21 × 30 cm). These specimens were then cut to the cone calorimeter size of 100 × 100 mm.

## 3. Results

The use of inorganic fillers for imparting flame-retardant properties on a wood surface mostly targets the isolation of the wooden mass from the flame. To do so, a high amount of inorganic filler is needed in order to compensate for the increasing flammability of the polymeric matrix that is used for a high-quality coating formation. At the same time, the increasing amount of the inorganic filler normally deteriorates the coating properties. In order to compromise the above-mentioned aspects, according to our approach, reactive organic additives that enable the dispersion of high amounts of inorganic fillers onto a water-based polymeric matrix were used. For the preparation of coatings, a polyacrylate-based polymeric matrix was used, named Vilacryl DS55K. As an aqueous system, this type of matrix tends to minimize air pollution in comparison with other kinds of matrices based on organic solvents. In addition, nanosized Mg (OH)_2_ from hydrolysis of the calcined minerals was used, as was presented in our previous work [18]. In order to improve the dispersibility of the Mg-based particles, epoxide reactive oligomers (ROs) were employed, which present multiple advantages as compatibilizers in comparison with the traditional ones. The ROs used in the present work were synthesized through free radical polymerization (FRP) of a hydrophilic monomer and another monomer bearing epoxide groups. The hydrophilic nature of the ROs contributes to the dispersion stabilization of the aqueous medium, whereas the epoxide groups are able to chemically link with the inorganic Mg-based particles. In previous studies, sulfonated polystyrene and the epoxide monomer glycidyl methacrylate have been used for the synthesis of ROs [21,22].

### 3.1. Characterization of Coatings at Lab Scale

At first, the polymer matrix Vilacryl DS55K was tested in terms of film formation and reaction-to-fire performance. Thus, the film of the polymer matrix was developed without huntite or additives. The film quality was excellent in all cases. LOI measurements are presented in Table 1. As it is observed, the polymeric matrix presented low flame retardancy, with an LOI value of 20.5. Then, the incorporation of huntite H5 into Vilacryl DS55K led to an increase in the LOI values from 20.5 to 28.0. The Vilacryl DS55K/Huntite H5 blend also exhibited excellent film quality, indicating a very good mix of the two materials.

**Figure 2:**
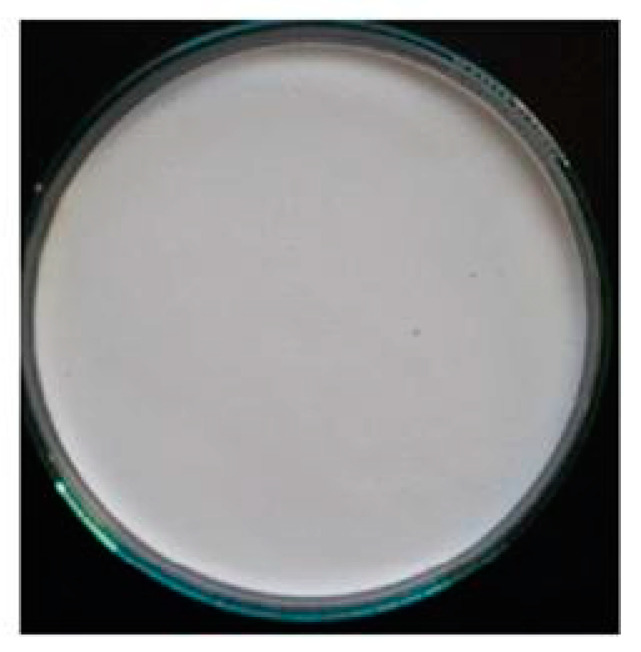
Photograph of the coating of sample WF-5.

With the introduction of ROs into the matrix/huntite mixture, the coating quality remained excellent, as shown in Figure 2, and even greater increases in the LOI values were observed. As it may be seen, the ROs played an important role when the matrix/huntite ratio was higher than 1. Even in the cases where this ratio was 0.1 (WF-5) and 0.18 (WF-7) and despite the fact that the matrix had a lower content in the blend, the coatings led to high LOI values. This is strong evidence of the fundamental function of the ROs in the coatings’ flame retardancy. In general, mixtures with low polymeric matrix content, high H5 huntite values and ROs between 14–23.6% led to higher LOI values. This may be attributed to the enhancement of the compatibility between the filler and the matrix owed to functionalization with the epoxide reactive oligomers (ROs).

In general, coatings with the epoxide oligomer P (SSNa-co-GMA20) (WF-5 to WF-9) presented high LOI values, except for WF-9, which had an LOI value of 28.0, similar to that of WF-2 without ROs. In the cases of WF-5, WF-7 and WF-8 coatings, LOI values were >78.0, >80.0 and 43.5, respectively. From the results, it seems that the ratio of Huntite H5/P (SSNa-co-GMA20) of 73.0/14.0% in WF-7 was optimal, such that a synergistic effect between the additive and the matrix occurred and thus led to a higher LOI value. Moreover, it is evident that a high RO loading, i.e., 40% ROs in WF-6, often acted as an inhibitor of the flame retardancy of the coatings. In the cases of WF-6 and WF-9, where the H5 content was constant at 50 %, LOI values were different since the coating WF-6 had an LOI value of 52.0, whereas the LOI for WF-9 was 28.0. This behavior may be owed to the different quantities of ROs (40% in WF-6 and 10% in WF-9), proving that the LOI enhancement in WF-6 with higher RO content was due to the synergistic effect between the additive and the matrix.

In order to confirm the beneficial effect of the ROs on the coatings’ flame retardancy, the homopolymer PSSNa was also applied for the coating formations Vilacryl DS55K /Huntite H5/PSSNa in WF-3 and WF-4, where the content of PSSNa was 14% and 40% of the total mass, respectively. The results showed that although the introduction of PSSNa gave an excellent film quality, it only slightly increased the reaction-to-fire performance of the Vilacryl DS55K/H5 mixture compared to P (SSNa-co-GMA). This is strong evidence of the role of GMA moiety in the mixture, as the addition of P (SSNa-co-GMA) ROs led not only to high-quality coatings but also to increased flame retardancy.

The excellent reaction-to-fire properties that P (SSNa-co-GMA20) provided to the mixtures may be attributed on the one hand to the high thermal stability of PSSNa and on the other hand to the better dispersion of the H5 particles after the modification of the hydroxyl and epoxy groups of the ROs, which results in the formation of a more compact and cohesive charcoal. In consequence, the very good dispersion of the H5 particles into the final coating prevented undesirable effects such as water pick up and swell, poor distribution and reduction in the physical properties of the composites.

It is worth noting that H5 releases amounts of water vapor (due to the presence of hydromagnesite in its structure at 12 wt%) and large amounts of CO_2_. The released gases dilute the oxygen and fuel gas concentrations. Moreover, huntite creates a compact and cohesive protective layer, thus preventing the fire from proceeding. The aforementioned synergistic effect of H5 with the ROs led to high values of LOI in the studied systems.

**Figure 3:**
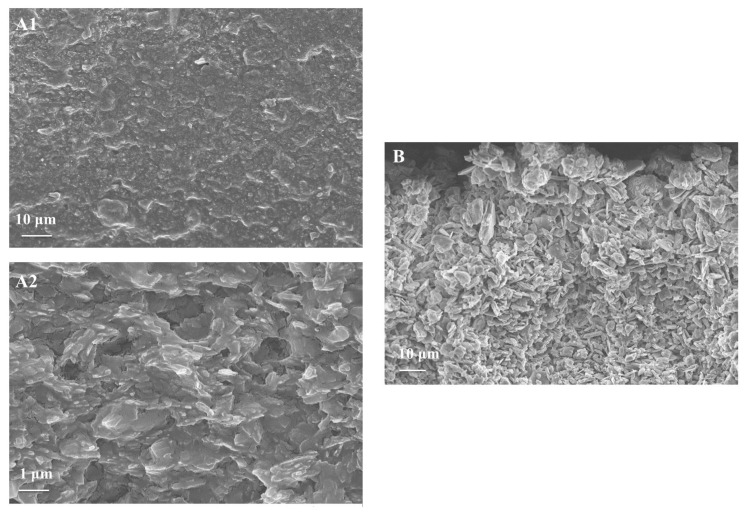
Cryofracture surfaces of SEM observation for Vilacryl DS55K/H5/P (SSNa-co-GMA20) coating, WF-7 (**A1**,**A2**), and Vilacryl DS55K/H5/PSSNa coating, WF-4 (**B**).

The dispersion ability of the epoxy-based RO, P (SSNa-co-GMA20), is clearly demonstrated by the SEM images presented in Figure 3, showing the N_2_-fractured cross-section of the Vilacryl DS55K/H5 coating with P (SSNa-co-GMA20) as the dispersant.

As may be seen from the SEM images (Figure 3A) at low magnification, the obviously good interaction between H5 and the additive P (SSNa-co-GMA20) led to very good coating dispersion. Figure 3B presents the Vilacryl DS55K/H5/PSSNa coating, where the low dispersibility of the mineral is obvious, which may be attributed to the absence of the epoxide groups. The enhanced quality of the coatings with the epoxide ROs is obvious in the SEM micrographs of Figure 3A due to the synergistic effect between the additive and the matrix. H5 particles were recognized through EDS elemental analysis, where Mg or Ca was detected. Moreover, it may be observed that the typical size of the H5 plate-like particles was on the ~1 μm scale. The shape of H5 particles is of substantial interest for flame-retardant applications, as it is known that platy morphology contributes to the reduction in heat and gas transfer rates and enhances polymer reinforcement [31,32] to reduce the rates of heat and gas transfer.

### 3.2. Application of the Modified Coatings on Wood Substrates and Characterization of the Treated Samples (Scale-Up)

The mixtures with Vilacryl DS55K, Huntite (H5 or H5-m) and the copolymer P (SSNa-co-GMA20) led to homogeneous and excellent-quality films presenting high LOI values. These systems were prepared on a larger scale for the coating of plywood substrates with dimensions 21 × 30 cm. The studied systems are shown in Table 2. The loading of the coatings onto the plywood substrates varied in the range of 382–1370 g/m^2^, while the coating specifications were selected in terms of the best results of flame retardancy obtained in the lab scale experiments (13% polymeric matrix, 73% Huntite and 14% ROs).

For the coating procedure, the layer-by-layer technique was applied either in one layer or in two layers. As mentioned by Qiu et al. [33], the layer-by-layer method is a simple, versatile and low-cost method with a number of advantages. It can be used for the preparation of multilayer films with controllable thickness, composition and function. Moreover, the layer-by-layer method may be applied at low concentrations and lead to thin and homogenous coatings. In the flame retardants applied in multilayers, we chose the upper layer in order to reduce the combustion process and avoid the modification of mechanical behaviors by the incorporation of flame retardants into the whole mixture [34,35]. Fire-retardant coatings prepared via the layer-by-layer method could be used as a potential substitute for additive flame retardants, exhibiting high flame-retardant efficiency, low environmental impact and low impact on the polymers’ intrinsic properties [36].

**Figure 4:**
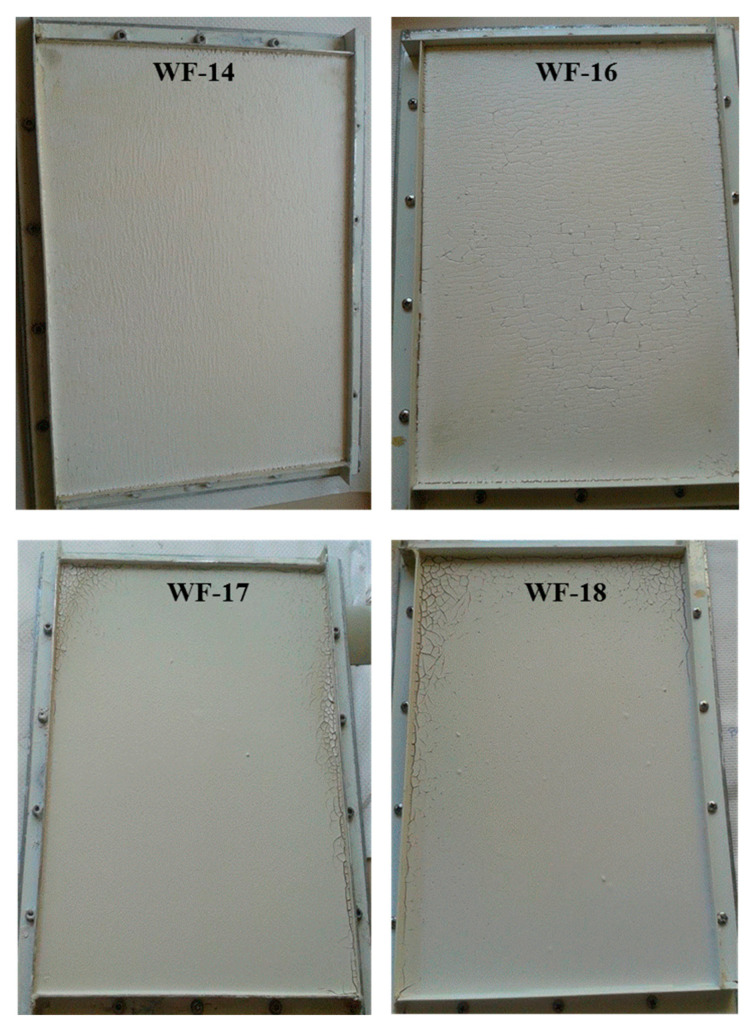
Photos of the optimized coatings with H5 and H5-m on wood substrates.

Based on this approach, for the one-layer coating application, a mixture of the Huntite-based H5 mineral or H5-m and the reactive oligomer P (SSNa-co-GMA20) was prepared in water, and the polymer matrix Vilacryl DS55K was subsequently added. After homogenization, the coating was spread on the plywood substrate. In the case of a two-layer coating, a mixture of the reactive oligomer P (SSNa-co-GMA20) and the mineral was prepared in water and applied as a first layer on the wood substrate. The system was left to dry at room temperature. After the evaporation of water, a second layer was subsequently applied over the wood substrate, consisting of the polymer matrix Vilacryl DS55K mixed with water, in order to obtain a less viscous solution. Figure 4 shows the macro-morphology study of the coatings covered on the plywood with the two-layer (WF-14) or the one-layer (WF-16, WF-17 and WF-18) coating application. It is obviously seen that no bubbling or peeling was observed, but a cracking effect appeared in some cases in WF-16, which was reduced to a great extent in WF-14, where no cracking appeared at all since the two-layer deposition was applied. The uniform smooth surface observed in most cases, as well as the whitening and non-transparency of the coatings, makes them good candidates to be used mostly as primers rather than finishing coatings.

### 3.3. Fire Test Results

**Figure 5:**
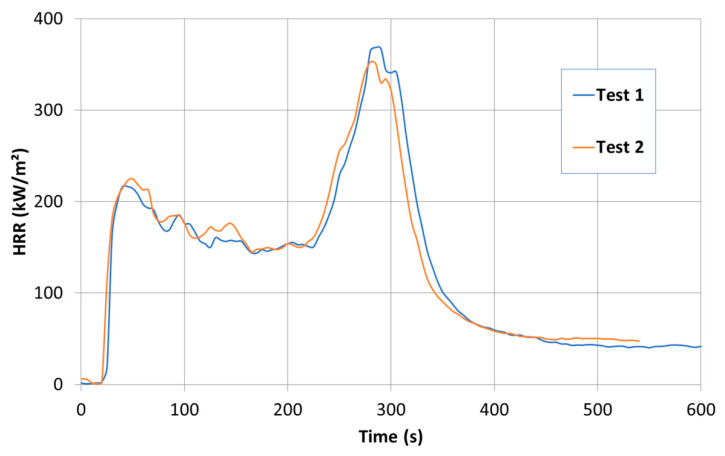
Heat Release Rate (HRR) curves vs. time in the cone calorimeter ISO 5660-1 for untreated plywood.

**Figure 6:**
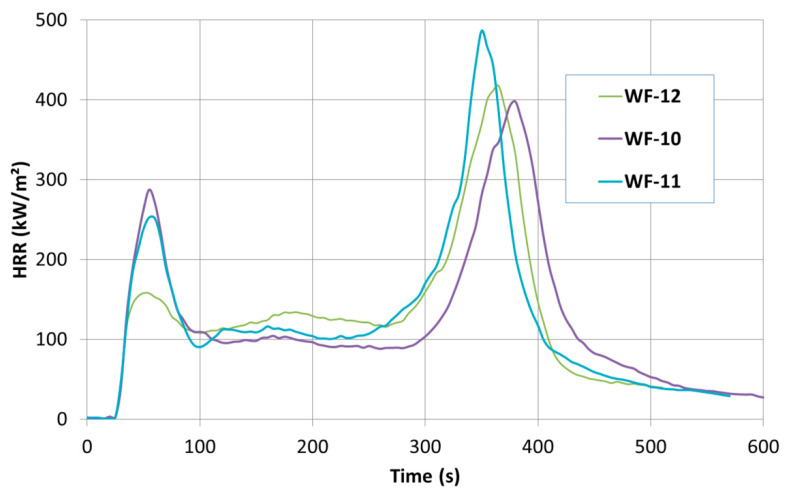
Heat Release Rate (HRR) curves vs. time in the cone calorimeter ISO 5660-1 for coatings WF-10, WF-11 and WF-12.

**Figure 7:**
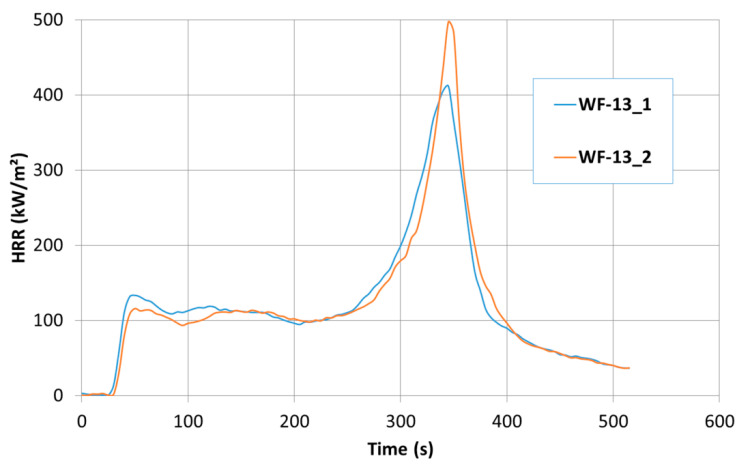
Heat Release Rate (HRR) curves vs. time in the cone calorimeter ISO 5660-1 for coating WF-13 (two tests).

**Figure 8:**
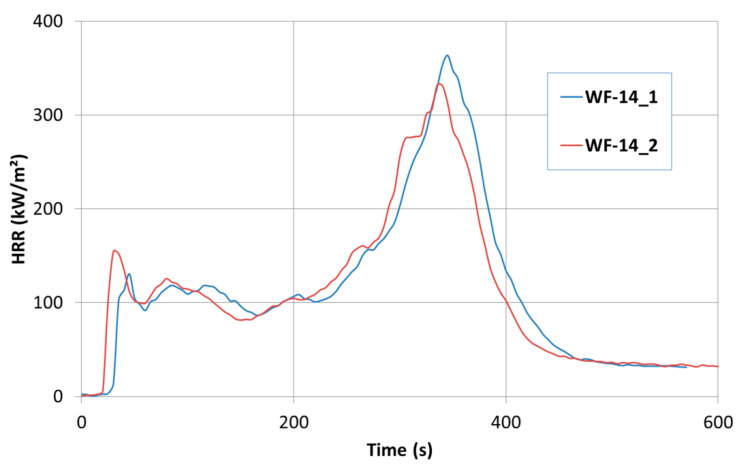
Heat Release Rate (HRR) curves vs. time in the cone calorimeter ISO 5660-1 for coating WF-14 (two tests).

**Figure 9:**
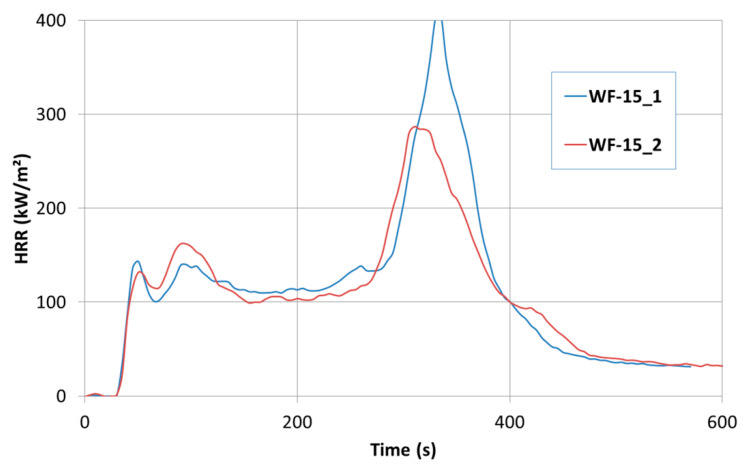
Heat Release Rate (HRR) curves vs. time in the cone calorimeter ISO 5660-1 for coating WF-15 (two tests).

**Figure 10:**
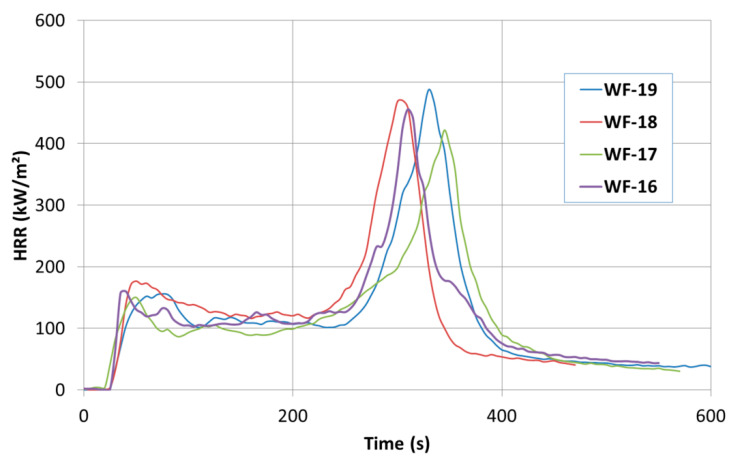
Heat Release Rate (HRR) curves vs. time in the cone calorimeter ISO 5660-1 for coatings WF-16, WF-17, WF-18 and WF-19.

Reaction-to-fire testing was carried out according to the cone calorimeter ISO 5660 at the heat flux 50 kW/m^2^ and complemented by predictions of possible reaction-to-fire classes according to the European system with Euroclass classification. The classification system for the reaction-to-fire properties of building construction products has been introduced in Europe as the Euroclass system [37] and consists of two sub-systems, one for construction products (wall and ceiling surface linings) and one for floorings. The classes for both systems are from A to F, where Al and A2 refer to non-combustible products. The desired Euroclass categories for flame-retardant coatings are B and C. The fire test results are summarized in Table 3, whereas the heat release rate vs. time curves are also given in Figure 5, Figure 6, Figure 7, Figure 8, Figure 9 and Figure 10.

The combustion process of wood may be divided into drying, pre-carbonization, carbonization and combustion, and the HRR depends on the structural stability of the wood component. In all diagrams, a first peak appears at <50 s, assigned to the decomposition of light volatile and partial hemicellulose, and a second peak appears at ~300–350 s, attributed to the remaining hemicellulose, cellulose and partial lignin, which are the stages of the sequential decomposition of wood [38].

The different techniques applied during the coating of the mixtures seemed to have an effect on the fire test results. More specifically, WF-10 and WF-11, which were prepared in one layer with loadings 1370 and 1010 g/m^2^, respectively, showed no difference in reaction-to-fire performance in comparison with raw wood, as they failed to change categories and remained in Euroclass D. Coating WF-12, which was prepared with H5 in one layer with loading 790 g/m^2^, was assigned Euroclass C. Coating WF-13 (two tests), with H5 in two layers and loadings ~900 g/m^2^, was assigned Euroclass B. Similar results appeared for coating WF-14 (two tests), which was prepared in two layers with loadings 370 and 770 g/m^2^ and assigned Euroclass B. In the same category, coating WF-15 (two tests) is included, with H5 in two layers and loadings 678–688 g/m^2^, which was also assigned Euroclass B. On the other hand, WF-16, WF-17, WF-18 and WF-19, prepared in one layer with the last two using H5-m instead of H5, changed categories to Euroclass C. Coatings WF-13, WF-14 and WF-15 not only managed to change categories but also were included in class B, which is the best possible for fire systems applied to wood. It was also observed that a high loading of the plywood substrates with the coating formulations often acted as an inhibitor of the flame retardancy of the coated plywoods. Moreover, it is important to note that the application technique seemed to be more crucial even than the loading of the plywood with the coating. In fact, the one-layer-coated plywoods showed lower flame retardancy than the two-layer-coated systems. This occurred probably because during the application of the fire to the calorimetry cone, the polymeric matrix, which consists mainly of hydrocarbons that form the upper layer, was burned first. Then, when the combustion proceeded to the additive layer with the huntite, the inorganic additive acted as a barrier to the wood, creating a compact and cohesive char. It also released large amounts of water vapor and/or CO_2_, which dilute the concentration of oxygen and combustible gases, delaying combustion.

Concerning Euroclass C, which was achieved with the nano-Mg (OH)_2_, H5-m, prepared by our group, the results are encouraging, since this is a desired Euroclass category for flame-retardant coatings. It has to be noted that magnesium hydroxide (MH) is included in the most effective halogen-free flame retardants for many applications, with its main action being endothermic dehydration at 300–340 °C [39,40]. The five-stage mechanism of MH is: endothermic decomposition, release of diluent gas, char formation, solid phase diluent and heat capacity of the mineral segment, which leads to the reduction in the energy amount available for polymer degradation [41]. However, the endothermic degradation of these hydroxides leads to non-cohesive and powder-like residues, which are not able to ensure an efficient protective barrier against fire. For this reason, the enhancement of the efficiency of MH by the development of new formulations was thoroughly examined through their partial substitution with synergistic additives such as reactive oligomers.

Epoxy resins are generally used in various types of materials, such as wood, fabric, glass and metal, and in a wide range of applications, such as electronics, construction, adhesives and protective and decorative coatings [42], due to their high versatility and intrinsic properties [43,44]. However, concerning flame-retardant applications, the combustion of epoxy resin may lead to high release rates of heat and smoke, causing serious consequences [45,46]. Thus, the modification of epoxy resins is crucial for improving flame retardancy while maintaining their good properties.

In the literature, epoxy resins are widely used with the addition of flame retardants due to their low cost, simple processing, low toxicity and high flame-retardant effects [47,48,49]. In the present work, we took advantage of the benefits of epoxy groups, and we synthesized the copolymer P (SSNa-co-GMA20), which combines the hydrophilic nature of SSNa with all the benefits of epoxy groups mentioned above. The specific copolymer was selected, as it combines the behavior of a charged polyelectrolyte and water solubility with the reactive epoxy functional groups, which are further used in the reaction with the hydroxyl groups of the hydromagnesite portion of the mineral H5 and H5-m, resulting in better dispersion, or for modification with the carboxyl groups of the polymeric matrix Vilacryl DS55K.

Table 4 shows the flame behavior of coatings reported in the literature in terms of LOI, time to ignition and HRR values. Some of the systems reported in Table 4 used epoxy resins or magnesium hydroxide particles for the preparation of flame-retardant coatings. Cong et al. [50] prepared hybrid composites of bio-based thin-ply carbon fiber prepreg and flame-retardant mats (E20MI). The inclusion of FR mats in the thin-ply bio-based prepreg revealed that the FR mat location can increase both the fire protection results and the mechanical properties. Miao et al. [51] compared the flammability of bio-based bis (2-methoxy-4-(oxiran-2-ylmethyl) phenyl)furan-2,5-dicarboxylate (EUFU-EP) with that of the commercial DGEBA cured with methyl hexahydrophthalic anhydride (MHHPA), showing higher flame retardancy.

Wan et al. [52] developed eugenol-based bifunctional epoxy resins (TPEU-EP) with improved flame retardancy thanks to the full aromatic ester backbone of TPEU-EP. Li et al. [53] linked epoxidized eugenol bio-epoxy (EPEU) with silicon-containing bridges with different lengths and chemical structures. It was found that the flammability of the prepared silicon-containing bio-epoxy resin was significantly improved in comparison to the commercial DGEBA. Among the halogen-free mixtures that Staszko et al. [54] prepared, containing epoxy resin, phosphorus compounds and Mg (OH)_2_, the mixtures Ep5 + 5 wt.% Mg (OH)_2_ (5M) and Ep5 + 10 wt.% Roflam F5 + 5 wt.% Mg (OH)_2_ (10F + 5M) showed the lowest heat release rates. Suihkonen et al. [55] observed a significant decrease (71%) in the burning rate of samples EP-10-N and EP-10-NU as a function of magnesium hydroxide (MDH) content compared to neat epoxy, indicating an improvement in flame-retardant properties upon MDH addition. Wang et al. [38] prepared ammonium thiocyanate (NH_4_SCN)-modified geopolymeric coatings and observed delayed flame propagation with the increasing content of NH_4_SCN up to 1 wt.%, since exceeding this dosage had a detrimental effect on flame retardancy.

Comparing the results of Table 4 with the results of other work, it may be concluded that the prepared coatings in Euroclass B with time to ignition between 32–38 (s) and HRR 115–150 kW/m^2^ have very good flame retardancy, which in some cases is even better than that observed in the literature. This is a significant observation for the wide application of flame-retardant composites combining water-soluble functional copolymers with high amounts of inorganic fillers in the building sector and other important fields.

## 4. Conclusions

Novel non-halogenated flame-retardant hybrid organic–inorganic coatings were prepared as potential materials for wood applications. The epoxide reactive oligomer P (SSNa-co-GMAx) was combined with H5 at a lab scale and with either H5 or H5-m in scale-up processing and subsequently dispersed in the polymer matrix VILACRYL DS55K. These systems may be applied for the protection of wood materials from fire in new buildings, restoration processes and furniture pieces. The system with 13% polymeric matrix, 73% Huntite and 14% ROs was tested in scale-up application, as it provided the best lab-scale coating quality and flame retardancy. Plywood substrates with dimensions 21 × 30 cm were coated with the layer-by-layer method at loadings in the range of 400–900 g/m^2^. Selected formulations showed high reaction-to-fire performance, reaching the criteria for Euroclasses B and C. Particularly, the coatings WF-13, WF-14 and WF-15 reached Euroclass B, with the best film quality observed in coatings WF-13 and WF-14 (in comparison to coating WF-15 with medium quality). Coating WF-14 could be more preferable, since it also needs a lower loading, 370 g/m^2^ in this case, in contrast to the other two coatings.

Our main aim was, through the functionalization of inorganic flame retardants with reactive oligomers (ROs), to enhance the compatibility between the filler and the matrix. The synergistic relationship between the ROs’ epoxide groups and the inorganic Mg-based particles avoids undesirable effects such as water pick up and swell, poor dispersion and distribution and a reduction in the physical properties of the composites.

## Figures and Tables

**Table 1:** Film quality and LOI results for coatings based on Vilacryl DS55K, H5 and ROs.

Code	Polymer Matrix Content (%)	H5 Content (%)	Additive Content (%)	LOI
Vilacryl DS55K				
WF-1	100	-	-	20.5 ± 0.5
Vilacryl DS55K /Huntite H5				
WF-2	50.0	50.0	-	28.0 ± 0.0
Vilacryl DS55K /Huntite H5/PSSNa				
WF-3	10.0	50.0	40.0	29.0 ± 2.0
WF-4	13.0	73.0	14.0	30.0 ± 0.0
Vilacryl DS55K /Huntite H5/P(SSNa-co-GMA20)				
WF-5	7.0	69.4	23.6	>78.0
WF-6	10.0	50.0	40.0	52.0 ± 2.0
WF-7	13.0	73.0	14.0	>80.0
WF-8	27.0	66.7	6.3	43.5 ± 1.5
WF-9	40.0	50.0	10.0	28.0 ± 0.0

**Table 2:** Characteristics of the coating formulations applied on wood substrates.

Code	Coating Specifications					Coating on Wood Substrate		
	Polymer Matrix (%)	Huntite (%)	Additive Content (%)	Huntite	Way of Coating	g/m^2^ Theoretical	g/m^2^ Experimental	Coating Quality ^a^
WF-10	13	73	14	H5	1 layer	1390	1370	+
WF-11	13	73	14	H5	1 layer	1028	1010	+
WF-12	13	73	14	H5	1 layer	800	790	+++
WF-13_1	13	73	14	H5	2 layers	910	900	+++
WF-13_2	13	73	14	H5	2 layers	912	904	+++
WF-14_1	13	73	14	H5	2 layers	400	370	+++
WF-14_1	13	73	14	H5	2 layers	800	770	+++
WF-15_1	13	73	14	H5	2 layers	600	678	++
WF-15_2	13	73	14	H5	2 layers	620	688	++
WF-16	13	73	14	H5	1 layer	400	382	+++
WF-17	13	73	14	H5	1 layer	600	569	++
WF-18	13	73	14	H5-m	1 layer	400	387	++
WF-19	13	73	14	H5-m	1 layer	600	588	++

^a^ +++: Excellent. ++: Medium. +: Low. Ø: No film.

**Table 3:** Reaction-to-fire test results for coatings and predicted possible European fire classes.

	Test Results from Cone Calorimeter, ISO 5660-1, at Heat Flux 50 kW/m^2^			
Code	Way of Coating	Time to Ignition (s)	Heat Release Rate, First Peak (kW/m^2^)	Possible Euroclass
WF-10	1 layer	31	287	D
WF-11	1 layer	36	258	D
WF-12	1 layer	31	158	C
WF-13_1	2 layers	36	125	B
WF-13_2	2 layers	36	115	B
WF-14_1	2 layers	32	143	B
WF-14_2	2 layers	34	139	B
WF-15_1	2 layers	37	137	B
WF-15_2	2 layers	38	150	B
WF-16	1 layer	31	160	C
WF-17	1 layer	24	150	C
WF-18	1 layer	31	177	C
WF-19	1 layer	30	156	C
	Untreated plywood (=standard substrate)			
Test 1		24	225	D
Test 2		28	216	D

**Table 4:** Comparative study of the literature.

System	LOI	Time to Ignition (s)	Heat Release Rate (kW/m^2^)	Reference
Rosin-based epoxy prepreg (AGMP-3600)	45.8	45	109	[50]
EUFU-EP/ MHHPA	-	200	291.3	[51]
TPEU-EP/ DDS	26.8	33	860	[52]
EPEU/ DDS/ Siloxane	28	28	662	[53]
5M	-	90	354	[54]
10F + 5M	-	72	353	[54]
EP-10-N	-	58	566	[55]
EP-10-NU	-	59	539	[55]
S2 (NH_4_SCN 1 wt.%)	-	102	248	[38]

## Data Availability

Not applicable.
